# Coronary pseudoaneurysm 1 week after complex percutaneous coronary intervention with drug‐eluting stent

**DOI:** 10.1002/ccr3.2910

**Published:** 2020-04-30

**Authors:** Mohammad Hasan Namazi, Mohammad Khani, Taraneh Faghihi Langroudi, Fatemeh Abedi, Mohammadreza Tabary, Isa Khaheshi

**Affiliations:** ^1^ Cardiovascular Research Center Shahid Beheshti University of Medical Sciences Tehran Iran; ^2^ Radiology Department Modarres Hospital Shahid Beheshti University of Medical Sciences Tehran Iran; ^3^ School of Medicine Tehran University of Medical Sciences Tehran Iran

**Keywords:** coronary angiography, coronary pseudoaneurysm, drug‐eluting stent, percutaneous coronary intervention

## Abstract

Pseudoaneurysm formation is a rare complication after complex PCI with drug‐eluting stents. Cardiologists and interventionist should be familiar with this rare complication after PCI and its management options.

## Graphical abstract

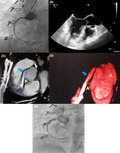

## 1 INTRODUCTION

During recent years, a great revolution has happened in interventional cardiology. This advancement is so remarkable in the diagnosis and treatment of complex lesions that had to undergo open cardiac surgery before the turning point in interventional cardiology. Endovascular management of coronary lesions is an appealing approach with lower procedural mortality and morbidity and high procedural success rates. Moreover, the management of probable life‐threatening complications after the percutaneous coronary intervention was a terrible catastrophe for interventionists. However, significant developments in equipment, skills, and imaging modalities have facilitated the management of these complications.

Coronary artery pseudoaneurysm has been described as a relatively rare event after vessel wall injury and is believed as an adverse consequence after drug‐eluting stent implantation, principally in complex lesions. Management of coronary pseudoaneurysm is a challenge in interventional cardiology, and there is no confirmed consensus regarding its treatment; so, each case should be considered individually regarding its specific condition.^1^, ^2^, ^3^, ^4^, ^5^

## 2 CASE PRESENTATION

A 55‐year old man referred to our hospital with the chief complaint of exertional chest pain of functional class II (FC II) in the last 4 weeks, which improved to FC III last week. He had a history of coronary artery bypass graft (CABG) surgery 2 years ago and was in good condition after the CABG. He was admitted with the impression of unstable angina and underwent coronary angiography, which showed patent left intramammary artery (LIMA) on LAD, occluded all saphenous grafts and chronic total occlusion (CTO) of native right coronary artery (RCA) and left circumflex artery (LCx).

**Figure 1:**
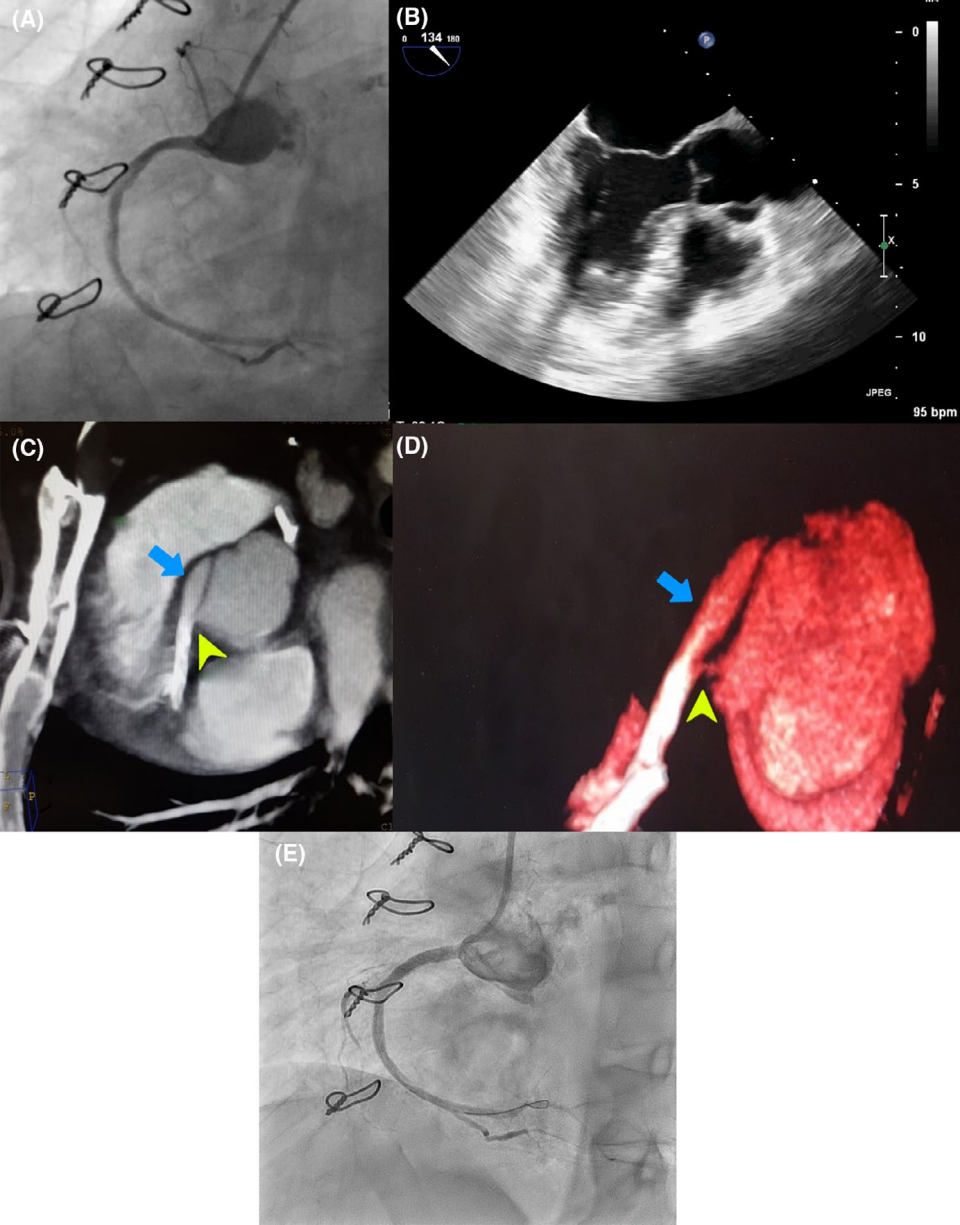
A, Right coronary injection showed a significant pseudoaneurysm at the proximal portion of RCA. B, Transesophageal echocardiography revealed thick intimal flap in the right sinus of Valsalva possessing flow within. C and D, Coronary CT angiography confirmed the presence of pseudoaneurysm at the proximal portion of RCA (blue arrows indicate pseudoaneurysm, and yellow arrowheads indicate the origin of pseudoaneurysm). E, Pseudoaneurysm was sealed off after the deployment of a covered stent at the ostium of RCA with acceptable final result

According to angiography results, he was a candidate for angioplasty of CTO on RCA, which was accomplished successfully with 3 drug‐eluting stents (DES). Due to the CTO lesion of the LCx artery, he was scheduled for a staged PCI on LCx 1 week later. Before starting the angioplasty of LCx, RCA was injected, which revealed a significant pseudoaneurysm at the proximal portion of RCA (Figure 1A, Video S1). According to angiography results, pseudoaneurysm did not seem to be connected with the aortic sinus of Valsalva. At this step, the interventionist decided to prepare the patient for coronary CT angiography and transesophageal echocardiography (TEE) to achieve more detailed information.

TEE showed thick intimal flap in the right sinus of Valsalva possessing flow within (Figure 1B). Coronary CT angiography was performed and confirmed the presence of pseudoaneurysm at the proximal portion of RCA (Figure 1C and D).

The patient was prepared for intravascular ultrasonography (IVUS) which showed local dissection at the proximal part of RCA. Finally, the interventionist decided to deploy a covered stent at the ostium of RCA. Before performing this plan, we deployed a balloon at the ostium of RCA during aortography. As an interesting result, the pseudoaneurysm disappeared after balloon deployment. Finally, we used a BeGraft covered stent (3.5*21 mm) at the ostium of RCA and was postdilated with NC balloon (4*12 mm) (Figure 1E). The pseudoaneurysm was sealed off, and the final result was acceptable. After the procedure, the patient had no remarkable complaints and was discharged with a good condition after 3 days of hospitalization.

## 3 DISCUSSION

Even though they have made a considerable revolution in terms of extremely low rates of restenosis in interventional cardiology, imperative safety concerns on the extensive usage of drug‐eluting stents (DES) have been discussed.^3^, ^6^

Coronary artery pseudoaneurysm has been described as a rare complication after drug‐eluting stents, particularly after implantation in complex lesions during PCI. These lesions have been reported to evolve 1 week to 4 years after DES implantation. Various mechanisms have been proposed as potential explanations for pseudoaneurysm development, including local inflammatory reaction, drug toxicity, infection, and acute vessel injury during the preliminary procedure.^4^, ^6^, ^7^, ^8^

Management of the coronary pseudoaneurysm is a challenging issue, and there is no standard guideline for the treatment; so, each case should be considered individually regarding its specific condition.^1^, ^5^, ^9^

We presented a challenging case of RCA pseudoaneurysm 1 week after DES implantation. At initial evaluation, it seemed to be connected with the aortic sinus of Valsalva according to TEE results. However, coronary CT angiography confirmed the diagnosis of the pseudoaneurysm. IVUS and deploying balloon at the ostium of RCA before coronary intervention affirmed the underlying pseudoaneurysm.

Coronary artery bypass surgery could be an acceptable treatment option for remarkable coronary pseudoaneurysm; however, the perioperative cardiac surgery risks would be an imperative limitation for this approach. On the other hand, PCI may be considered as an appropriate alternative approach for the coronary pseudoaneurysm in patients who do not desire to undergo surgical therapy. It is also associated with shorter hospitalization in elective settings. Endovascular treatment of coronary artery pseudoaneurysm via covered stent is a promising approach with lower procedural mortality and morbidity rates, concurrently solving the aneurysm‐related symptoms with high procedural success rates.^1^, ^2^, ^5^

This case report highlights the importance of the coronary artery pseudoaneurysm formation, as a rare complication after complex PCI with drug‐eluting stents. Cardiologists and interventionist should be familiar with this rare complication to select the best treatment choice.

## CONFLICT OF INTEREST

None declared.

## AUTHOR CONTRIBUTIONS

MHN: served as the main author and contributed to data acquisition and manuscript preparation. MK: contributed to data acquisition and performing laboratory tests. TFL: contributed to data acquisition in terms of performing cardiac evaluations. FA: contributed to manuscript writing. MT: contributed to manuscript writing, editing, and final revision of the manuscript and the submission process. IK: served as the corresponding author and designed and supervised all the aspects. Each author: contributed sufficiently and met the criteria for authorship.

## Supporting information

Video S1
